# The influence of job characteristics toward intention to pursue sales career mediated by feelings

**DOI:** 10.3389/fpsyg.2022.953645

**Published:** 2022-11-09

**Authors:** Siti Ngayesah Ab Hamid, Nadzirah Rosli, Roshayati Abdul Hamid, Che Aniza Che Wel

**Affiliations:** Faculty of Economics and Management, Universiti Kebangsaan Malaysia, Bangi, Malaysia

**Keywords:** sales career, job characteristics, feelings, intention, students

## Abstract

The sales profession has suffered from negative perception and misconception. Despite a huge opportunity in this industry, several reasons have been highlighted as deterrents to job applicants from applying for a career in sales including the unethical practices, low prestige, and uncertainty of the job. This study examines the influence of job characteristics and feelings on intention to pursue a sales career among undergraduate and postgraduate students. A cross-sectional study was conducted with 251 questionnaires analyzed using Partial-Least Square-Structural Equation Modeling (PLS-SEM). The findings suggest that job characteristics and feelings have a favorable impact on intention. Job characteristics, on the other hand, influence feelings, and feelings mediate the relationship between job characteristics and intention. This study contributes to the body of knowledge by examining feelings as one of the constructs influencing sales career intention, and provides several implications to employers and business instructors to develop effective strategies to spark student interest in sales-related careers.

## Introduction

In 2020, the world has been hit by the unprecedented event of COVID-19, causing the worst economic downturn in Malaysia since the last decline in 1998. The implementation of various Movement Control Order (MCO) to contain the spread of COVID-19 has led to the increase in number of job losses and subsequently rise in the country’s numbers of unemployment by 200 thousand to record 718.1 thousand persons in 2020. Fresh graduates in particular are not an exception as they are equally affected by the pandemic. Competition to get a job among graduates is getting more challenging with the presence of more unemployed persons who lost their jobs during the pandemic (DOSM, 2020).

In Malaysia, a study done in 2021 reported that sales and marketing positions are the most demanded job by the organization. However, this job does not seem to be appealing for most of the graduates. Previous research reported that students have conflicting perceptions toward a career in sales and sales jobs (Karakaya et al., 2011). The earlier studies reported that students have negative perceptions toward sales careers, which is associated with poor image. The misconception of sales career has resulted in low participation of university graduates in the sales force.

Literature on sales demonstrate that society still does not embrace sales as a profession, with the evidence from studies of more than 60 years reflecting negative attitudes toward sales as career (Mason, 1965; Paul and Worthing, 1970; Joonas and Caballero, 2009; Bahhouth et al., 2014). Although sales is a critical component for the success of many organizations, many college seniors are reluctant to consider a career in sales (Manning et al., 2010). Furthermore, Weber (2015) found that college students have unfavorable perceptions of sales which result in less interest to pursue a career in sales. However, another research has shown that approximately 70% of those receiving degrees in marketing will choose sales as their initial career position (Kutscher, 1990; Weilbaker, 2001; Michaels and Marshall, 2002; Simon, 2006). This may be due to the nature of this generation who are confident, prefer less-structured environments, and appreciate being in control, which is offered in sales career (Kim et al., 2009).

The term “sales” is defined as all activities involved in selling a product and/or service to a consumer or business. In practice, it means so much more. There is a great deal of efforts undertaken toward closing a deal; the processes start from sourcing for prospects, to building relationships and offering solutions to customers. These tedious processes of sales determine a unique job characteristic which contributes to a negative or positive feeling toward a sales career.

The sales industry builds a connection/link between businesses and their potential buyers of products and services. A salesperson will inform the customers about product features and benefits and they will also answer customers’ questions, negotiate terms and price, and take orders. In short, because of the nature of its work, a sales job has unique job characteristics that may influence candidates whether to favor or not to favor those jobs. In addition, those unique job characteristics will influence a person’s feelings toward this job. Thus, the goal of this study is to investigate the influence of sales job characteristics toward the intentions to pursue a sales career. The relationship between feelings about selling and intention to pursue a sales career will also be examined.

Specifically, the study aims to achieve these research objectives:

1. To examine the influence of job characteristics toward the intention to pursue a sales career.
2. To investigate the relationship between feelings about selling and intention to pursue a sales career.
3. To determine the mediating role of individual feelings between job characteristics and intention to pursue a sales career.

To the best of our knowledge, there is a lack of studies that investigated the interaction between job characteristics and feelings toward sales careers. In addition, most of the previous studies investigated job characteristics and feelings in the context of employees’ workplace. While in this study, the respondents are among university students who have no experience in sales careers but they have an initial knowledge and feeling toward the sales job from the program curriculum. Therefore, they are the potential employees to enter the workplace as a salesperson. Therefore, the present study aims to fill the gaps in the literature to understand whether job characteristics and feeling have an impact on student intention to join the sales careers. In order to do this, we analyze the survey data using partial-least square-structural equation modeling (PLS-SEM) from the data collected *via* a survey of 251 university students who are currently pursuing a bachelor degree and master’s in business. The proposed model analyzed two variables, namely job characteristics and feelings, which influence intention to pursue sales careers.

The structure of this paper is arranged as follows. The first section reviewed relevant previous literature and proposed the research hypotheses. Next, the methodology of the study is discussed, followed by the presentation of analysis and results. The final sections highlight the study contributions and limitations of the study as well as suggest areas for future research.

## Literature review

Career selection is an important starting point as it is a crucial aspect of people’s lives. The profession a person chooses reveals what kind of a life he/she will lead to, his/her ambition in life, identity, the way to utilize his/her potential, self-respect, social support, financial gain, and the way he/she spends the time (Unguren and Huseyinli, 2020). Previous studies found that there are a low number of qualified employees in the sales industry (Unguren and Huseyinli, 2020). This scenario shows that sales jobs are less favorable for job seekers. Because of the unique characteristics of a sales job, the industry nowadays requires salespeople who are unique, vivid, ambitious, competent, and well-prepared to quickly adjust to the constantly shifting business environment (Pettijohn, 2009). In meeting this growing demand of skilled salespeople, businesses have turned to universities in order to look for potential sales recruits (Wiles and Spiro, 2004). It is more appealing to recruit college students due to the fact that they are perceived as young, knowledgeable, and able to learn and react quickly (Dubinsky, 1981). Hence, college graduates are often viewed as “work ready” and are able to survive in the ever-changing sales landscape (Peltier et al., 2014).

However, recruiters generally find that it is more complicated to persuade students to apply or interview for sales positions, and it is even harder to get them to pursue a job, particularly in the sales field (Dubinsky, 1981). In reality, a sales career is seen as rewarding and fulfilling, but students usually have a negative impression of selling, and hence, their intention to engage in a sales career is indeed low (Karakaya et al., 2011). More than often, it is due to the misperception and negative stereotypes about selling which are often propagated by the mass media (Lee et al., 2007). In addition, the uncertainty of the job (Ehtiyar and Unguren, 2008) is also cited as another reason that hinders graduates from pursuing sales jobs.

Empirical evidence from previous studies revealed that career intentions are influenced by a variety of factors such as interest (Rahman et al., 2020), intrinsic motivation, extrinsic motivation, and professional capacity or self-awareness of one’s own professional capacity (Zhang et al., 2020), personal interest that mediated by one’s soft skills, self-efficacy, and social influence (Muhamad et al., 2020). Only individuals who have self-efficacy have the courage to choose sales as a career because they have the ability to see their strengths and weaknesses, always be positive, and believe in themselves. The direct effects of perceived sales knowledge, perceptions of salespeople, and perceptions of the selling profession were significant and positive on students’ intent to pursue a sales career (Cummins and Peltier, 2020). Not only that, other studies also found that sales career intention is also influenced by education exposure (Nielson and Cummins, 2019). For example, internship satisfaction may play a significant role in determining the students’ intention to develop a career in the industry. There are several factors that influence students’ satisfaction with internship experience and their career intention; these include internship achievements, mentorship and assessment, interpersonal relationships, compensation, company features, company’s internship program, and curriculum requirements (Qu et al., 2021). Despite these findings, offering good financial reward is not sufficient in attracting salespeople, but how a salesperson feels about their job, its role requirements, and outcomes are very important to make them ready to work and contribute positively to the company (Jaramillo et al., 2013).

In the past decades, the majority of literature on personal selling and its career appeal largely consists of research among U.S. and U.K. students (Fournier et al., 2014). Students outside the Anglosphere were often ignored (Ballestra et al., 2017). Nonetheless, there are a few articles addressing the non-Western countries, especially in Malaysia, which explored the key factors that influence students’ perceptions of sales-related careers, including academic performance, gender (Sohail, 2015), personal development, communication skills (Rahman et al., 2014), inner circle (Rahman et al., 2014), income (Rahman et al., 2014), attitude, subjective norm, and knowledge (Mokhlis et al., 2022). However, there is little insight available that looks particularly into factors such as job characteristics and feelings. Exploring these issues from an international perspective may be important for several reasons, including the fact that different countries may perceive and view sales careers in different ways.

### Job characteristics and the intention to pursue sales career

In the early years, the nature of the workplace was characterized by routine processes that often led to demoralization and cause boredom to employees. Hence, stemming from this issue, the Job Characteristic Model (JCM)/theory was introduced by Hackman and Oldham (1976). The model was based on the idea that certain job characteristics will motivate employees due to the intrinsic joy they receive in performing the job activities (Richard Hackman and Oldham, 1976). According to Syptak et al. (1999), employees will appreciate their job and they will be driven to perform effectively if they find their job characteristics entertaining and meaningful. There are five core dimensions in the JCM, namely, skill variety, task identity, task significance, autonomy, and feedback. These dimensions affect three critical psychological states (i.e., meaningfulness, responsibility, and knowledge of results), which subsequently lead to personal and work outcomes, including work performance, motivation, and satisfaction (Hackman and Oldham, 1976; Cavanagh et al., 2020). According to Becherer et al. (1982), sales jobs’ characteristics are generally viewed as an important factor that determines salespeople’s commitment, satisfaction, motivation, and performance. This concurs with some empirical evidence from previous studies which found that job characteristics is related to the positive outcome such as job satisfaction (Cavanagh et al., 2020), organizational commitment, and OCB (Akingbola and Van Den Berg, 2019), as well as negatively related to turnover intention (Ghosh et al., 2015).

Traditionally, in the 20^th^ century, the concept of sales is merely “selling of products and services” (Leigh and Marshall, 2001; Ballestra et al., 2017). In the past, sales were associated with negative perceptions and it is often characterized as being unethical (Lee et al., 2009), low in prestige (Bristow et al., 2006), benefiting uneducated buyers, and exaggerating product benefits (Bristow et al., 2006). The selling situation itself is also filled with uncertainty and interpersonal conflict which requires persistence and owned self-motivation (Dubinsky et al., 1986). However, there has been continuous change on how sales are organized and performed. Leigh and Marshall (2001) described these as “the plethora of changing conditions.” There are numerous reasons that contribute to the changes including technological advances, competitive intensity, globalization, heightened stress on the customer-seller relationship, and development of the selling team (Ingram, 2004). These have caused critical challenges to the sales organizations, especially concerning the required skills and competencies of the salesperson. Besides, these changes also break the traditional view and the landscape of sales jobs.

In recent years, contrary to the past, sales is more toward building and maintaining long-term relationship (Leigh and Marshall, 2001; Ballestra et al., 2017; Handley et al., 2017). Therefore, salesperson in the present are characterized as individuals who are adaptable, resilient, and self-driven (Lassk et al., 2012), possess emotional wisdom (Bagozzi et al., 2010), exhibit a thorough understanding on organizations, behavior, information collection, market research, and sales forecasting (Allen et al., 2014), and well-versed in technologies (Walker et al., 2009). This results in the birth of a new generation of youthful, clever, highly-trained, ambitious, and customer-oriented individuals (Lassk et al., 2012).

Sales careers are typically commission-based and profit-driven, thus stress and pressure are common among salespeople who need to achieve the sales quota. Previous research has shown that some people are intrigued in jobs that provide challenges and other job characteristics that push them to perform under pressure (Wiesenfeld et al., 2014; Han et al., 2018; Kabil et al., 2018). Besides, research by Handley et al. (2017) has shown that both males and female students believe that one particular feature of sales jobs that outshine others is the excitement. Furthermore, they found that Asian students viewed sales careers as more exciting, innovative, and fun, compared to Australian students. Therefore, in line with the findings from literature, the study hypothesized that:

H1: Job characteristics influence the students’ intention to pursue a sales career

### Sales job characteristics and feelings toward sales career

Karakaya et al. (2011) indicate that perceptions of sales jobs and salespeople influence individuals’ feelings toward selling and intention to pursue a sales career. This is because job perceptions may predict individual psychological feelings (Moore, 2000). Perception is a process of how an individual interprets and responds to the information into something meaningful, while feeling is used to describe sensation through experience or perception (Shouse, 2005). Sometimes graduates enter the salesforce with no clear understanding of what the role will entail, often leading to higher turnover (Boles et al., 2012). The salesperson stereotypes are shown to be consistently negative across cultures and this negatively impacts student recruitment (Lee et al., 2007) and consumer behavior (Babin et al., 1995). Therefore, the marketing campaigns need to be designed to educate students about the importance of sales as a career (Inks and Avila, 2018). The campaign must focus on the sales job attributes or characteristics which will assist educators to address students’ misconceptions of the sales profession. According to Ballestra et al. (2017), sales job attributes such as job outcomes and job requirements have a significant impact on students’ feelings toward selling. But they found that the feelings toward selling depend on students’ perception of sales job outcomes rather than sales job requirements. Students perceive a sales job mostly as a venture that can provide career growth opportunities followed by esteemed personality and it makes them feel satisfied and valued (Ballestra et al., 2017). In addition, this perception will help students to feel more motivated and confident about the success of the sales profession (Inks and Avila, 2018). Thus, the study hypothesized that;

H2: Job characteristics influence the students’ feeling towards a sales career.

### Feeling toward sales career and The intention To pursue sales career

Both sales careers and salespeople have been largely viewed as a low reputation job this perception has not been addressed so far (Avlonitis and Panagopoulos, 2010). The academic literature has addressed this issue since the 1950s, primarily by acquiring a better grasp on the perceptions of students on personal selling (Sojka et al., 2000). Apart from that, the sales job is also often seen negatively and remained least favored by students over time (J.W. Peltier et al., 2014; Sari, 2014).

According to Sandelands (1988), feeling is “a response to a stimulus. It is an evaluation of, or judgment about, a specific object or thing.” The feelings are triggered by emotions and formed through personal experiences, beliefs, memories, and thoughts associated with that particular emotion. In other words, a feeling is the by-product of the brain processing an emotion and attaching a certain meaning to it (LeDoux, 2012). During the late 1950s to early 1970s, it is found that students generally have a negative feeling toward selling and they also have several misconceptions about sales (Ballestra et al., 2017). Later research also found that one-third of the respondents have a negative impression about selling (Muehling and Weeks, 1988), which is often associated with door-to-door activities or undesirable personal traits, such as obnoxious, out of style, and pushy.

From 1980 to 1990, it was found that students’ preferences for a sales career are heavily influenced by the characteristics of financial benefits and decision-making power, rather than the functionality of a sales job (Swenson et al., 1993). Later, students were reported to feel a more positive view of personal selling as a career (Dubinsky, 1981; Muehling and Weeks, 1988). Nowadays, students’ feelings toward sales careers appear to be similar to views during the 1960s and the 1970s, as they perceive sales careers rather positively (Karakaya et al., 2011; Peltier et al., 2014). Research by Karakaya et al. (2011) has revealed that students’ feelings toward sales are indeed positive, including that it provides a sense of accomplishment, excitement, value, productivity, interesting features, and personal satisfaction, which is consistent with prior findings (Dubinsky, 1981; Muehling and Weeks, 1988; Bristow et al., 2006; Handley et al., 2017).

Literature also shows that feeling toward selling positively influences the intention to pursue a sales career among students (Karakaya et al., 2011; Ballestra et al., 2017). Majority also expressed that working in a profession they enjoy or love is also more productive, gratifying, and significant than the monetary value (Morrison, 2021). Thus, this proves that a positive feeling will lead to a positive work performance. Hence, in line with these findings, the study hypothesized that:

H3: Feelings towards selling will influence students’ intention to pursue a sales career

Despite the fact that the nature of selling has dramatically changed over the years, there is still concern that the positive perceptions do not translate into positive inclination toward a sales career (Karakaya et al., 2014), particularly among business students. Job characteristics should be controlled to test attraction to career employment (Kjeldsen and Jacobsen, 2013). Scholars have mostly adopted job characteristics to characterize specific career choices and have conducted a large number of related studies based on choices (Alhomoud et al., 2019). A good fit between career and job characteristics greatly strengthened employees’ work motivation and had a significant influence on career success and choices (Alhomoud et al., 2019). Previous studies reveal that people are more likely to choose jobs in different sectors when they have opportunities to satisfy their altruistic work values through relational jobs (Choi, 2017). Furthermore, the job characteristic model highlighted that various job characteristics will influence job perception and feelings, which further affect individuals’ values, behavior, and attitude (Chen, 2014). Hence, it is important to explore whether students with different feelings choose different career paths when considering job characteristics which may affect students’ career intentions. Hence, the study hypothesized that:

H4: The relationship between job characteristics and students’ intention to pursue a sales career is mediated by their feelings towards sales career.

## Methodology

### Sampling design and data collection procedure

A cross-sectional research design was utilized with self-administered questionnaires distributed online *via* WhatsApp application among undergraduate and postgraduate business faculty students at the Universiti Kebangsaan Malaysia. Online data collection was used as this study was conducted during the Covid-19 pandemic which restricted people’s movement and pushed all classes to be conducted *via* the online platform. Non-probability convenience sampling technique was employed in collecting the data as it is the best alternative due to the time and movement constraint during the pandemic.

In a period of 3 weeks, 254 questionnaires were collected. However, only 251 questionnaires were valid for further analysis after excluding three questionnaires that suffered from a straight lining problem.

The majority of the respondents were female (59.4%) and the highest age group was 19–24 years old (76.1%). Most of the respondents were Malay (61.4%), while 62.5% of the respondents were Muslims. Some of the respondents work full-time while studying (44.6%) and most of the respondents were full-time students (73%). Majority of the respondents (33.9%) were from marketing majors while the other respondents were from other business majors such as management (28.3%) and accounting (14.7%). Half of the respondents (53.4%) have family members working in the sales and marketing field, and most of the respondents (70.5%) have experience in selling. Most of the respondents (75.7%) have experience enrolling in a sales or marketing course and 68.1% of the students have a plan to start their own business.

### Research instrument

The measurement items used in this study were adapted from existing validated instruments as shown in Table 1. Items for sales job characteristics were adapted from Dubinsky (1981), while items for feelings and intention to pursue sales career were adapted from Karakaya et al. (2011). Five-point Likert scale was used to measure all the constructs. The questionnaires also include questions on the respondent’s profile such as gender, age, ethnicity, religion, program majoring, and sales job-related questions such as experience and sales course enrolment. The questionnaire was prepared and distributed in English as English is the second language in Malaysia and is understood by the majority.

**Table 1:** Sample of measurement items.

Variables	Source	No of items	Sample of items
Job Characteristics	Dubinsky (1981)	5	Use of college education Good remuneration Dynamic/exciting
Feelings	Karakaya et al. (2011)	4	Selling is personally satisfying Selling is interesting Selling gives a sense of accomplishment
Intention	Karakaya et al. (2011)	4	I am very interested in pursuing a professional sales-related career after obtaining my degree Obtaining a sales support position would interest me Obtaining a position in sales is a priority for me after graduation.

### Data analysis

Data were analyzed using SPSS and Partial-Least Square-Structural Equation Modeling (PLS-SEM) technique. PLS-SEM has been widely used in various models where the relationship between constructs has been previously defined (e.g.: (Saura et al., 2022) as it allows understanding on the influence of each constructs. Several studies on career intention have also used PLS-SEM technique to analyze the data collected using survey method (Amaning et al., 2020; Beuk et al., 2022).To use it, a two-step approach was applied, starting with the analysis on the validity and reliability of the constructs known as the measurement model, and followed by the assessment of the structural model. However, before employing PLS-SEM analysis, a Harman’s single-factor test was conducted to examine for common method variance. Result of factor analysis performed on all the items showed that the shared variance was 35.83%, which is less than the 50% threshold. This shows that no domination of a single factor exists, indicating that common method bias does not significantly influence the findings.

## Result

### Measurement model

In order to assess the reflective measurement model proposed in this study, convergent validity and discriminant validity are evaluated. Table 2 shows the factor loadings, AVE, and CR that are used to test convergent validity. Most of the factor loadings are above the recommended value of 0.708, except for one indicator which is Feel3 which has been deleted. In the meantime, the AVE result for all constructs met the satisfactory level of >0.5 (Bagozzi and Yi, 1988), while the composite reliability (CR) value also met the acceptable value of >0.7 (Gefen et al., 2000).

**Table 2:** Assessment of internal consistency and convergent validity.

Construct	Item	Loading	CR	AVE
Job characteristics	Job1	0.767	0.905	0.657
	Job2	0.841		
	Job3	0.851		
	Job4	0.852		
	Job5	0.735		
Feelings	Feel1	0.519	0.742	0.509
	Feel2	0.575		
	Feel3	*Deleted*		
	Feel4	0.963		
Intention	Intention1	0.887	0.947	0.817
	Intention2	0.921		
	Intention3	0.895		
	Intention4	0.911		

In addition, Table 3 shows the result of discriminant validity examined using Heterotrait Monotrait (HTMT) technique (Henseler et al., 2015). The values are lower than HTMT_.85_ (Henseler et al., 2015), indicating that discriminant validity for the constructs in is study is established.

**Table 3:** Heterotrait monotrait (HTMT) criterion for discriminant validity.

	Job characteristics	Feelings	Intention
Job characteristics			
Feelings	0.178		
Intention	0.717	0.306	

### Structural model

A lateral collinearity issue may mislead the findings as it can disguise a strong causal effect that exists in the model (Kock and Lynn, 2012). As such, to ensure no lateral collinearity issue in the structural model, inner VIF values were examined. The values in Table 4 show that all the VIF values are less than 0.5 indicating the non-existence of collinearity.

**Table 4:** Assessment of structural model.

Path Hypotheses	Std Beta	Std Error	*t*-value	*p* value	Decision	VIF	f^2^	*R* ^2^	Q^2^
H1: Job Characteristics = > Intention	0.623	0.047	13.174	0.000	*Supported*	1.023	0.701	0.458	0.368
H2: Job characteristics = > Feelings	0.149	0.088	1.683	0.050	*Supported*	1.000	0.023	0.018	0.003
H3: Feelings = > Intention	0.189	0.185	3.156	0.001	*Supported*		0.064		
H4: Job Characteristics = > Feelings = > Intention	0.028	0.016	1.786	0.037	*Supported*				

**Figure 1:**
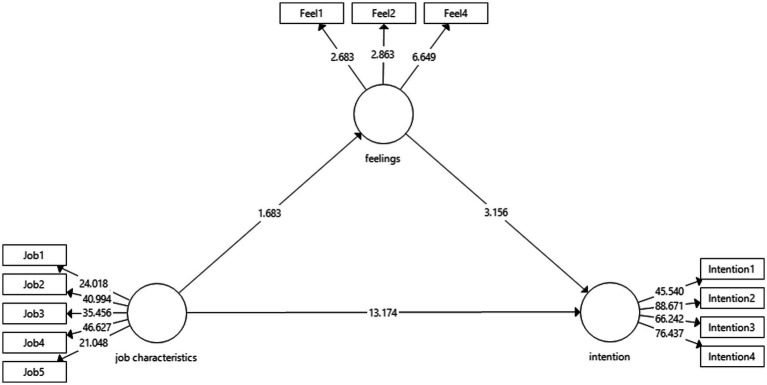
Structural model.

The bootstrapping result of 5,000 resamples also is shown in Table 4 and Figure 1. For the direct relationship, the result shows that hypotheses 1, 2, and 3 on the influence of job characteristics toward intention (*β* = 0.623, *p* < 0.01), job characteristics toward feelings (*β* = 0.149, *p* < 0.05), and feelings toward intention (*β* = 0.189, *p* < 0.01), respectively, are supported. Meanwhile, the result shows that job characteristics have significant indirect effects toward intention *via* feelings (*β* = 0.028, *p* < 0.05). Thus, it could be concluded that the mediation effect is statistically significant.

The in-sample explanatory power *R*^2^ for intention (0.458) is moderate, while the *R*^2^ (0.018) for feelings is weak (Hair et al., 2019). Other than that, the f^2^ value of job characteristics (0.701) indicates a substantial effect size in generating the R^2^ for intention (Chin, 2010), while feelings indicates small effect size for intention. The predictive relevance (Q^2^) of the model shows that the values of intention (0.368) and feelings (0.003) are larger than zero, indicating that the model has sufficient predictive relevance.

## Discussion

The present study investigated the effects of job characteristics and feelings toward intention to pursue a sales career. Based on previous literature, four hypotheses were proposed and tested and the result shows that all the hypotheses are supported.

The first hypothesis predicted that job characteristics would have an influence on students’ intention to pursue a sales career. The PLS results revealed that there is a positive relationship between job characteristics and intention to pursue sales careers. This finding indicates that the nature of the job that fits the individuals is important in determining individuals’ intention to apply for that particular job. This finding is consistent with Wiesenfeld et al., 2014; Han et al., 2018; and Kabil et al., 2018, which shows a significant impact of job characteristics toward intention to apply for various jobs including nursing and teaching.

The second hypothesis predicted the influence of job characteristics toward students’ feelings on sales careers. The PLS-SEM results indicate that there is a positive relationship between job characteristics and students’ feelings toward sales careers. This is in agreement to Ballestra et al. (2017) which stated that sales job attributes have an impact on students feeling toward sales career. The result is also consistent with Moore (2000) who says that perception toward job characteristics could predict an individual psychological feeling.

Hypothesis 3 predicts that feeling toward selling will influence students’ intention to pursue sales careers. The analysis of the data reveals that there is a positive relationship between feeling and students’ intention to pursue sales careers. Previous research have shown that positive feelings toward selling including the sense of accomplishment, excitement, productivity, and personal satisfaction could influence students intention to pursue a sales career (Karakaya et al., 2011). Moreover, similar to this study, Beuk et al. (2022) also found that feelings toward sales career indeed able to predict intention.

The last hypothesis proposed the mediation role of feelings in the relationship between job characteristics and intention. The PLS-SEM results indicate that feeling plays a significant role as a mediator. This is in line with Chen, 2014, statement that job characteristics could influence job perception and feelings which, in turn, able to affect individual’s intention and behavior. Thus, it could be concluded that desirable job characteristics lead to a positive feeling toward sales jobs and increase students’ intention to pursue the sales job.

## Conclusion

Choosing a career in sales is rewarding as it has a clear career path and has the potential to earn a high remuneration. However, not many graduates are inclined toward this career (Karakaya et al., 2011), which is due to various factors including misperception, negative stereotypes (Lee et al., 2007), and uncertainty of the job (Ehtiyar and Unguren, 2008). To understand this situation in Malaysia, this study was conducted to investigate factors influencing intention to pursue a sales career among Malaysian graduates from two angles which are job characteristics and feelings. The study found that all the hypotheses proposed are supported. In addition, the study also found that feelings play a mediating role in the relationship between job characteristics and intention to pursue sales careers. As sales jobs demand a breadth of skills including consultative, knowledge-based, and greater understanding of customers, the job characteristics dimensions seem attractive since the candidate can utilize a variety of skills with significant task job autonomy. The nature of the job itself will also lead to a positive feeling toward the jobs thus leads to a greater intention to pursue the sales careers.

### Theoretical implications

Several implications could be concluded from this study. Theoretically, the study contributes to the field by including feelings as one of the constructs influencing sales job intention, which is lacking in the literature. Second, the study also tested the role of feelings as a mediator in the relationship between job characteristics and intention. However, the result shows that as compared to feelings (*β* = 0.189), job characteristics play a bigger role (*β* = 0.623) in influencing sales job intention. This finding indicates the prominent role of job criteria in attracting good candidates to join the sales force. The study also shows that job characteristics have an impact on feelings, signifying the importance of creating good perception toward sales job so that positive feelings would emerge among future prospects. Despite the superiority of job characteristics toward intention as compared to feelings, the significant relationship shows that feelings indeed play some role in influencing intention. As studies including feelings as one of the construct are limited (Cummins et al., 2016; Beuk et al., 2022), future studies could explore this construct in various contexts and in other models to test its role.

### Practical implications

Practically, the study provides several implications to employers and business lecturers to develop effective strategies to spark student interest in sales careers. First, employers and the industry should ensure that the sales career itself is attractive in terms of the job characteristics, especially from the aspect of remuneration, dynamic and exciting nature of the work, and the relationship with co-workers. Not only that, the negative connotations related to sales careers such as unethical and low prestige also should be eliminated. In order to do so, business lecturers and employers could share the good prospects of the job in terms of building customer relationship, the usage of technology, and the chance to experience a sense of achievement, as the job itself is challenging and tough but rewarding (Wiesenfeld et al., 2014; Han et al., 2018; Kabil et al., 2018). Second, as feelings toward selling is related to perception toward the job, a campaign to educate students on the importance of sales career (Inks and Avila, 2018) and outcome of the job in terms of career growth opportunities would also be valuable (Ballestra et al., 2017) and would help students to see the prospects of success in the sales profession. Other than that, in order to ensure that students’ feelings toward selling could be translated to intention to pursue sales career, more positive feeling should be created by relating the sales job with the feel of excitement, personally satisfying, interesting features, and productivity (Bristow et al., 2006; Handley et al., 2017), while the misconceptions that associate sales career with door-to-door activities and undesirable personal traits, such as out of style and pushy, should be eradicated.

### Limitations and future research

Despite achieving its objectives, there are some limitations that need to be considered for future studies. First, the data were collected using convenience sampling method; hence, cautions should be made in generalizing the findings outside of the sample. Second, data were also collected among business students in UKM only, thus limiting its generalizability to the other students in Malaysia. Third, the study focuses on two factors affecting intention only. Future studies may examine other possible factors such as job outcome and personality traits. In addition, demographic characteristics such as ethnicity and gender also could be investigated. Furthermore, future research can further explore job characteristics by utilizing qualitative methods.

## Data availability statement

The original contributions presented in the study are included in the article/supplementary material, further inquiries can be directed to the corresponding author.

## Ethics statement

Ethical review and approval were not required for the study on human participants in accordance with the local legislation and institutional requirements. Written informed consent for participation was not required for this study in accordance with national legislation and institutional requirements.

## Author contributions

SA conducted the analysis, interpreted the data, and wrote the discussion and conclusion section. NR and RA conducted and wrote the literature review section. CC wrote the introduction section. All authors contributed to the study design and data collection and read the final manuscript before submission.

## Funding

The study is funded by MPOB-UKM-2021-012 grant.

## Conflict of interest

The authors declare that the research was conducted in the absence of any commercial or financial relationships that could be construed as a potential conflict of interest.

## Publisher’s note

All claims expressed in this article are solely those of the authors and do not necessarily represent those of their affiliated organizations, or those of the publisher, the editors and the reviewers. Any product that may be evaluated in this article, or claim that may be made by its manufacturer, is not guaranteed or endorsed by the publisher.

## References

[ref1] Akingbola K. Van Den Berg H. A. (2019). Antecedents, consequences, and context of employee engagement in nonprofit organizations. Rev. Public Pers. Admin. 39, 46–74. doi: 10.1177/0734371X16684910

[ref2] Alhomoud F. K. AlGhalawin L. AlGofari G. AlDjani W. Ameer A. Alhomoud F. (2019). Career choices and preferences of Saudi pharmacy undergraduates: a cross sectional study. Saudi Pharm. J. 27, 467–474. doi: 10.1016/j.jsps.2019.01.009 31061614 PMC6488871

[ref3] Allen C. Kumar P. Tarasi C. Wilson H. (2014). Selling sales: factors influencing undergraduate business students’ decision to pursue sales education. J. Mark. Educ. 36, 94–104. doi: 10.1177/0273475314537279

[ref4] Amaning N. Anim R. O. Kyere A. Peprah-Amankona G. (2020). Determinants of career intentions of accounting students. Int. Bus. Res. 13:14. doi: 10.5539/ibr.v13n12p14

[ref5] Avlonitis G. J. Panagopoulos N. G. (2010). Selling and sales management: an introduction to the special section and recommendations on advancing the sales research agenda. Ind. Mark. Manag. 39, 1045–1048. doi: 10.1016/j.indmarman.2009.12.006

[ref6] Babin B. J. Boles J. S. Darden W. R. (1995). Salesperson stereotypes, consumer emotions, and their impact on information processing. J. Acad. Mark. Sci. 23, 94–105. doi: 10.1177/0092070395232002

[ref7] Bagozzi R. P. Belschak F. Verbeke W. (2010). The role of emotional wisdom in salespersons’ relationships with colleagues and customers. Psychol. Mark. 27, 1001–1031. doi: 10.1002/mar.20370

[ref8] Bagozzi R. P. Yi Y. (1988). On the evaluation of structural equation models. J. Acad. Mark. Sci. 16, 74–94. doi: 10.1007/BF02723327

[ref9] Bahhouth V. Spillan J. Karsaklian E. (2014). Are students driven by negative or positive perception about sales profession in France? Euro. J. Bus. Soc. Sci. 3, 16–32.

[ref10] Ballestra L. V. Cardinali S. Palanga P. Pacelli G. (2017). The changing role of salespeople and the unchanging feeling toward selling: implications for the HEI programs. J. Mark. Educ. 39, 176–189. doi: 10.1177/0273475317724842

[ref11] Becherer R. C. Morgan F. W. Richard L. M. (1982). The job characteristics of industrial salespersons: relationship to motivation and satisfaction. J. Mark. 46, 125–135. doi: 10.1177/002224298204600413

[ref12] Beuk F. Weidner K. L. Houser L. M. (2022). Extending the validity and reliability of the intent to pursue a sales career scale. J. Mark. Educ. 1–21. doi: 10.1177/02734753221085031

[ref13] Boles J. S. Dudley G. W. Onyemah V. Rouziès D. Weeks W. A. (2012). Sales force turnover and retention: a research agenda. J. Pers. Sell. Sales Manag. 32, 131–140. doi: 10.2753/PSS0885-3134320111

[ref14] Bristow D. N. Gulati R. Amyx D. Slack J. (2006). An empirical look at professional selling from a student perspective. J. Educ. Bus. 81, 242–249. doi: 10.3200/JOEB.81.5.242-249

[ref15] Cavanagh T. M. Kraiger K. Henry L. K. (2020). Age-related changes on the effects of job characteristics on job satisfaction: a longitudinal analysis. Int. J. Aging Hum. Dev. 91, 60–84. doi: 10.1177/0091415019837996, PMID: 30897924

[ref16] Chen C. F. (2014). The influences of university interns’ job characteristics, work value, and job performance. Rev. de Cercetare şi Int. Soc. 47, 204–219.

[ref02] Chin W. W. (2010). “How to Write Up and Report PLS Analyses” in Handbook of Partial Least Squares: Concepts, Methods and Applications Heidelberg: Springer. 655–690.

[ref17] Choi Y. (2017). Work values, job characteristics, and career choice decisions: evidence from longitudinal data. Am. Rev. Public Adm. 47, 779–796. doi: 10.1177/0275074016653469

[ref18] Cummins S. Loe T. Peltier J. W. (2016). Using sales competition videos in a principles of marketing class to improve interest in a sales career. J. Adv. Mark. Educ. 24, 16–21.

[ref19] Cummins S. Peltier J. W. (2020). Understanding students’ decision-making process when considering a sales career: a comparison of models pre-and post-exposure to sales professionals in the classroom. J. Pers. Sell. Sales Manag. 41, 1–16.

[ref20] DOSM (2020). Graduate statistics 2020, Department of Statistics Malaysia Official Portal. Available at: dosm.gov.my (Accessed 25 April 2022).

[ref21] Dubinsky A. J. (1981). Perceptions of the sales job: how students compare with industrial salespeople. J. Acad. Mark. Sci. 9, 352–367. doi: 10.1007/BF02729877

[ref22] Dubinsky A. J. Howell R. D. Ingram T. N. Bellenger D. N. (1986). Salesforce socialization. J. Mark. 50, 192–207. doi: 10.1177/002224298605000405

[ref23] Ehtiyar V. R. Unguren E. (2008). A research of determination of hopelessness and anxiety levels of students studying tourism education in terms of attitudes of education. J. Int. Soc.Res. 1, 159–188.

[ref24] Fournier C. Chéron E. Tanner J. F. Jr. Bikanda P. J. Wise J. A. (2014). A Cross-cultural investigation of the stereotype for salespeople: professionalizing the profession. J. Mark. Educ. 36, 132–143. doi: 10.1177/0273475314536399

[ref25] Gefen D. Straub D. Boudreau M. C. (2000). Structural equation modeling and regression: guidelines for research practice. Comm. Assoc. Inform. Sys. 4:7.

[ref26] Ghosh P. Rai A. Chauhan R. Gupta N. Singh A. (2015). Exploring the moderating role of context satisfaction between job characteristics and turnover intention of employees of Indian public sector banks. J. Manag. Dev. 34, 1019–1030. doi: 10.1108/JMD-10-2014-0138

[ref27] Hackman J. R. Oldham G. R. (1976). Motivation through the design of work: test of a theory. Organ. Behav. Hum. Perform. 16, 250–279. doi: 10.1016/0030-5073(76)90016-7

[ref01] Hair J. F. Risher J. J. Sarstedt M. Ringle C. M. (2019). When to use and how to report the results of PLS-SEM. European Business Review 31, 2–24. doi: 10.1108/EBR-11-2018-0203

[ref28] Han S. W. Borgonovi F. Guerriero S. (2018). What motivates high school students to want to be teachers? The role of salary, working conditions, and societal evaluations about occupations in a comparative perspective. Am. Educ. Res. J. 55, 3–39. doi: 10.3102/0002831217729875

[ref29] Handley B. Shanka T. Rabbanee F. K. (2017). From resentment to excitement - Australasian students’ perception towards a sales career. Asia Pac. J. Mark. Logist. 29, 1178–1197. doi: 10.1108/APJML-07-2016-0133

[ref30] Henseler J. Ringle C. M. Sarstedt M. (2015). A new criterion for assessing discriminant validity in variance-based structural equation modeling. J. Acad. Mark. Sci. 43, 115–135. doi: 10.1007/s11747-014-0403-8

[ref31] Ingram T. N. (2004). Future themes in sales and sales management: complexity, collaboration, and accountability. J. Mark. Theory Pract. 12, 18–28. doi: 10.1080/10696679.2004.11658528

[ref32] Inks S. A. Avila R. A. (2018). An examination of high schools’ students’ perceptions of sales as an area to study in college, and factors influencing their interest in sales as a career to pursue after college. J. Mark. Educ. 40, 128–139. doi: 10.1177/0273475317752451

[ref33] Jaramillo F. Mulki J. P. Boles J. S. (2013). Bringing meaning to the sales job: the effect of ethical climate and customer demandingness. J. Bus. Res. 66, 2301–2307. doi: 10.1016/j.jbusres.2012.03.013

[ref34] Joonas K. Caballero R. (2009). Mexican student’s attitudes towards personal selling: an exploratory investigation. AIMS Int. J. Manag. 3, 221–240.

[ref35] Kabil N. S. Allam G. G. Abd El-Geleel O. M. (2018). Motivational reasons for choosing dentistry as a professional career & factors affecting specialty choice among final year dental students. Fut. Dent. J. 4, 308–313. doi: 10.1016/j.fdj.2018.04.002

[ref36] Karakaya F. Quigley C. Bingham F. (2011). A cross-national investigation of student intentions to pursue a sales career. J. Mark. Educ. 33, 18–27. doi: 10.1177/0273475310389151

[ref04] Karakaya F. Quigley C. Bingham F. Hari J. Nasir A. (2014). Business students’ perception of sales careers: Differences between students in Switzerland, Turkey, and the United States. Journal of Education for Business 89, 13–19. doi: 10.1080/08832323.2012.740520

[ref37] Kim H. J. Knight D. K. Crutsinger C. (2009). Generation Y employees’ retail work experience: the mediating effect of job characteristics. J. Bus. Res. 62, 548–556. doi: 10.1016/j.jbusres.2008.06.014

[ref38] Kjeldsen A. M. Jacobsen C. B. (2013). Public service motivation and employment sector: attraction or socialization? J. Public Adm. Res. Theory 23, 899–926. doi: 10.1093/jopart/mus039

[ref39] Kock N. Lynn G. S. (2012). Lateral collinearity and misleading results in cariance-based SEM: an illustration and recommendations. J. Assoc. Inf. Syst. 13, 546–580. doi: 10.17705/1jais.00302

[ref40] Kutscher R. E. (1990). Outlook 2000: the major trends. OCCUP. Outlook Q. 34, 3–7. 10113224

[ref41] Lassk F. G. Ingram T. N. Kraus F. Mascio R. D. (2012). The future of sales training: challenges and related research questions. J. Pers. Sell. Sales Manag. 32, 141–154. doi: 10.2753/PSS0885-3134320112

[ref42] LeDoux J. (2012). Rethinking the emotional brain. Neuron 73, 653–676. doi: 10.1016/j.neuron.2012.02.004, PMID: 22365542 PMC3625946

[ref43] Lee N. Beatson A. Garrett T. C. Lings I. Zhang X. (2009). A study of the attitudes towards unethical selling amongst Chinese salespeople. J. Bus. Ethics 88, 497–515. doi: 10.1007/s10551-009-0302-y

[ref44] Lee N. Sandfield A. Dhaliwal B. (2007). An empirical study of salesperson stereotypes amongst UK students and their implications for recruitment. J. Mark. Manag. 23, 723–744. doi: 10.1362/026725707X230018

[ref45] Leigh T. W. Marshall G. W. (2001). Research priorities in sales strategy and performance. J. Pers. Sell. Sales Manag. 21, 83–93.

[ref46] Manning G. L. Reece B. L. Ahearn M. (2010). Selling Today. 11th Edition. Upper Saddle River, NJ: Prentice Hall, Inc.

[ref47] Mason J. L. (1965). The low prestige of personal selling. J. Mark. 29, 7–10. doi: 10.1177/002224296502900402

[ref48] Michaels R. E. Marshall G. W. (2002). Perspective on selling and sales management education. Mark. Educ. Rev. 12, 1–11. doi: 10.1080/10528008.2002.11488780

[ref49] Mokhlis S. Nik Hussin N. S. Nizam N. Z. Mohd Noor N. A. Muslim N. A. (2022). Predicting Malaysian university students intent to pursue retailing career: applicability of theory of planned behavior. Int. J. Prof. Bus. Rev. 7, 1–26. doi: 10.26668/businessreview/2022.v7i1.277

[ref50] Moore J. E. (2000). Why is this happening? A causal attribution approach to work exhaustion consequences. Acad. Manag. Rev. 25, 335–349. doi: 10.2307/259017

[ref51] Morrison S. (2021). Life’s too short: 4 reasons to do what you love for a living., Business News Daily. Available at: https://www.businessnewsdaily.com/7995-reasons-to-do-what-you-love.html (Accessed 4 April 2022).

[ref52] Muehling D. D. Weeks W. A. (1988). Women’s perceptions of personal selling: some positive results. J. Pers. Sell. Sales Manag. 8, 11–20. doi: 10.1080/08853134.1988.10754477

[ref53] Muhamad H. San O. T. Katan M. B. H. Ni S. W. (2020). Factors influencing the personal interest, and behavioural intention to become an accountant in Malaysia. Int. J. Aca. Res. Bus. Soc. Sci. 10, 773–785. doi: 10.6007/IJARBSS/v10-i2/7005

[ref54] Nielson B. Cummins S. (2019). Recruiting sales students: the value of professionals in the classroom. Mark. Educ. Rev. 29, 65–74. doi: 10.1080/10528008.2018.1537717

[ref55] Paul G. W. Worthing P. (1970). A student assessment of selling. South. J. Bus. 5, 57–65.

[ref56] Peltier J. W. Cummins S. Pomirleanu N. Cross J. Simon R. (2014). A parsimonious instrument for predicting students’ intent to pursue a sales career: scale development and validation. J. Mark. Educ. 36, 62–74. doi: 10.1177/0273475313520443

[ref57] Pettijohn C. E. (2009). An exploratory analysis of sales career desirability: an MBA perspective. Aca. Edu. Lead. J. 13, 35–48.

[ref58] Qu H. Leung X. Y. Huang S. (. S.). He J. (2021). Factors affecting hotel interns’ satisfaction with internship experience and career intention in China. J. Hosp. Leis. Sport Tour. Educ. 28:100311. doi: 10.1016/j.jhlste.2021.100311

[ref59] Rahman M. K. Jali M. A. Ali M. A. Al-Mamun A. (2014). Determinants of students interest in sales job as potential profession: an empirical investigation in Malaysia. J. Mark. Manag. 2, 1–15.

[ref60] Rahman M. K. Mohamad M. Khan A. H. (2014). What motivational factors influence students’ interest in sales career? An empirical investigation in Malaysia. IOSR J. Bus. Manag. 16, 73–79. doi: 10.9790/487x-16327379

[ref61] Rahman R. S. A. R. A. Othman N. Talkis N. B. M. (2020). The influence of attitude, interest, teachers and peers on entrepreneurial career intention. Univ. J. Educ. Res. 8, 78–88. doi: 10.13189/ujer.2020.082110

[ref62] Sandelands L. E. (1988). The concept of work feeling. J. Theory Soc. Behav. 18, 437–457. doi: 10.1111/j.1468-5914.1988.tb00509.x

[ref63] Sari O. Y. (2014). ‘Students perception on career in sales: the challenge for educators and industrial users’, in Teaching and Learning in the 21st Century: Challenges for Lecturers and Teachers, pp. 263–267.

[ref64] Saura J. R. Ribeiro-Soriano D. Palacios-Marqués D. (2022). Adopting digital reservation systems to enable circular economy in entrepreneurship. Manag. Decis. doi: 10.1108/MD-02-2022-0190

[ref65] Shouse E. (2005). Feeling, emotion, affect. M/C J. 8. doi: 10.5204/mcj.2443

[ref66] Simon B. (2006). The paper (money) chase. Sales Mark. Manag. 42, 37–40.

[ref67] Sohail M. S. (2015). Student attitudes towards careers in sales in Australia and Malaysia: a Cross-cultural analysis. SSRN Electron. J. 28, 1–13. doi: 10.2139/ssrn.2650369

[ref68] Sojka J. Z. Gupta A. K. Hartman T. P. (2000). Student perceptions of sales careers. Am. J. Bus. 15, 55–64. doi: 10.1108/19355181200000006

[ref69] Swenson M. J. Swinyard W. R. Langrehr F. W. Smith S. M. (1993). The appeal of personal selling as a career: a decade later. J. Pers. Sell. Sales Manag. 13, 51–64.

[ref70] Syptak J. M. Marsland D. W. Ulmer D. (1999). Job satisfaction: putting theory into practice. Fam. Pract. Manag. 6, 26–30.

[ref71] Unguren E. Huseyinli T. (2020). The moderating effect of student club membership on the relationship between career intention in the tourism sector and post-graduate employability anxiety. J. Hosp. Leis. Sport Tour. Educ. 27:100265. doi: 10.1016/j.jhlste.2020.100265 32982582 PMC7508706

[ref72] Walker I. Tsarenko Y. Wagstaff P. Powell I. Steel M. Brace-Govan J. (2009). The development of competent marketing professionals. J. Mark. Educ. 31, 253–263. doi: 10.1177/0273475309345197

[ref73] Weber L. (2015). Why it’s so hard to fill sales job. Wall Street J. 6

[ref74] Weilbaker D. C. (2001). Why a career in sales (careers in professional selling). Waco, TX: Hankamer School of Business, Baylor University, Center for Professional Selling.

[ref75] Wiesenfeld L. Abbey S. Takahashi S. G. Abrahams C. (2014). Choosing psychiatry as a career: motivators and deterrents at a critical decision-making juncture. Can. J. Psychiatr. 59, 450–454. doi: 10.1177/070674371405900808, PMID: 25161070 PMC4143302

[ref76] Wiles M. A. Spiro R. L. (2004). Attracting graduates to sales positions and the role of recruiter knowledge: a reexamination. J. Pers. Sell. Sales Manag. 24, 39–48. doi: 10.1080/08853134.2004.10749015

[ref77] Zhang T. Li L. Bian Y. (2020). Final-year pharmacy undergraduate students’ career intention and its influencing factors: a questionnaire study in Northwest China. BMC Med. Educ. 20, 1–10. doi: 10.1186/s12909-020-02342-8 PMC7640686 33148230

